# A classification of specific movement skills and patterns during sprinting in English Premier League soccer

**DOI:** 10.1371/journal.pone.0277326

**Published:** 2022-11-11

**Authors:** Paul Caldbeck, Thomas Dos’Santos

**Affiliations:** 1 Sports Science Department, Sportlight Technology LTD, Oxford, United Kingdom; 2 Department of Sport and Exercise Sciences, Musculoskeletal Science and Sports Medicine Research Centre, Manchester Metropolitan University, Manchester, United Kingdom; 3 Manchester Institute of Sport, Manchester Metropolitan University, Manchester, United Kingdom; Sport Sciences School of Rio Maior - Politechnic Institute of Santarem, PORTUGAL

## Abstract

The aim of this study was to quantify and contextualize sprinting actions (≥ 7.0 m/s) of English Premier League (EPL) soccer match-play with respect to the movement skills and patterns employed. Video footage (3.2.6, Premier League DVMS, ChyronHego) and raw video-based locomotor coordinates of 901 sprint efforts from 10 matches of an EPL soccer team (2017–2018), were evaluated using the Football Sprint Movement Classification System pertaining to transition, initiation, and actualisation of sprint movement skills and patterns. The results from a one-way ANOVA or independent t-test revealed that, generally, most sprinting actions begun from a linear initiation position compared to lateral or rear (63%, *d =* 5.0–5.3, *p* < 0.01), without a change of direction (COD) (48%, *d* = 4.9–5.6, *p* < 0.01) compared to lateral, front-back, back-front, and from forward travelling (linear and diagonal) transition movements (68%, *d* = 2.1–5.7, *p* < 0.01) compared to lateral or rear. Additionally, most sprints were initiated with a rolling acceleration (66%, *d* = 3.2, *p* < 0.01) compared to explosive acceleration, often performed with a degree of curvature (86%, *d* = 7.2, *p* < 0.01) compared to linear, with torso rotation (62%, *d* = 2.7, *p* < 0.01) compared to no rotation, and typically end with an action such as duelling with an opponent or involvement with the ball (49%). Additionally, the sprint movement characteristics proportions slightly differed across playing positions. Overall, this study confirms that sprints during EPL soccer matches are initiated from and performed with a variety of different movement skills and patterns in relation to different sport-specific outcomes. This data can be used to assist in the development of more effective physical preparation programmes, inform position-specific contextualized sprinting drills to achieve better specificity and potential transfer of training, while also informing speed testing protocols.

## 1. Introduction

Locomotor movements such as high-velocity sprinting (≥ 7.0 m/s) are of a specific interest in soccer [1], with a plethora of literature investigating the sprint frequencies and distances of match-play [2–5]. Soccer is intermittent in nature [6], with the majority of running distance covered at lower, submaximal intensities [2–5]. However, brief periods of high-intensity explosive sprinting actions occur in soccer [6], and despite their reduced frequency and distances, sprinting is linked to decisive moments and performance, such as goal scoring, assisting, and defensive scenarios during match-play [7,8]. Importantly, distances covered sprinting have been reported to be increasing in EPL soccer [2,3], and are projected to rise over the next 10 years [9], whilst there is evidence indicating the ability to repeat sprint efforts and accumulate greater sprint distances are associated with match outcomes in the Bundesliga [10–12]. Moreover, high-velocity sprinting is also a common hamstring strain injury mechanism in soccer with negative implications [13]. Collectively, this highlights the importance for soccer players to be able to sprint effectively to improve performance and mitigate injury risk in soccer [14].

While insights and monitoring of sprint frequency and distances is indeed important in soccer, there is currently limited information regarding the situational and contextual sprinting demands of soccer, in particular how the sprints (i.e., movement skills and pattern) occur during match-play [15–17]. A greater understanding of sprinting match demands following an integrated approach [16,18] would allow practitioners to be able to further increase the specificity of their practice, assessment of sprinting, and ultimately enhance subsequent transfer of training [6,15]. Previous distance-based and frequency data in soccer ultimately lacks the intricate information around ‘how’ the sprints are performed (i.e., movement skills and patterns) and sprinting distance is specifically accumulated, and such an approach can be potentially too reductionist when quantifying the true demands of soccer match-play [6,15,17]. Only by fully understanding the movements associated with sprinting in soccer can practitioners truly prepare their players for the physical demands of soccer matches to enhance performance and mitigate injury risk.

Assuming that sprinting in soccer is solely linear, as distance and frequency data may suggest, without knowledge of its intricacies is potentially erroneous [19] and could be detrimental to the physical preparation and assessment strategies of soccer players [20]. It has been documented that soccer players perform arced runs, swerves, and curved sprints during soccer match-play [19,21,22], whereby curved sprinting can promote different neuromuscular, mechanical, and bioenergetic demands on the inside and outside legs during such actions [20,23,24]. Moreover, due to the visual scanning requirements associated during soccer (i.e., location of attackers, defenders, ball, environment) [20,25], and continuous changes in locomotor activity during sequences of match-play [20–22], generally over 360° [20,22], it is important to understand the transition, initiation, and actualization movement patterns of sprint actions during soccer (Table 1) [26], For example, sprints in soccer could be performed from different transition movements (e.g., linear, lateral, jockeying etc; Table 2), initiated from various starting positions (e.g., linear, lateral, rear etc.; Table 2) or from a COD, performed in a straight line or with some degree of curvature, potentially with some form of torso orientation for visual scanning purposes [6,26]. Additionally, these high-intensity actions could be performed for different attacking and defensive purposes with or without the ball, such as penetrating ‘runs in behind’, ‘overlapping runs’, ‘covering runs’, ‘pressing runs’ or ‘interceptions’ [6,16,17]. While studies have recently quantified physical-tactical high-intensity running demands (≥ 5.5 m/s) [16,17,27], to our best knowledge, no study has comprehensively quantified and described the sprinting (≥ 7.0 m/s) demands of EPL soccer match-play with respect to the movement skills and patterns with contextual classifications.

**Table 1:** The detailed sub-category descriptions from the Football Sprint Movement Classification System.

Main category	Sub-category	Description
**Transition**	Transition Movement	The movements completed immediately prior to the sprint effort.
**Initiation**	Starting Position	The position from which the sprint effort begins.
	Change Of Direction	The presence of any change of direction from the transition movement to the beginning the sprint effort
**Actualisation**	Acceleration	The type of acceleration used to complete the sprint effort
	Maximum Velocity	The direction of the sprint effort
	Torso Orientation	The existence of any dissociation of the torso
	Action During	Any possible action completed during the sprint effort
	Action End	Any possible action completed at the end of the sprint effort

**Table 2:** Football Sprint Movement Classification System, detailing all categories and action descriptions.

Main Category	Sub-Category	Action	Description
Transitional	Transition Movement	Static	*No movement of the feet*
		Jockeying	*Shuffling and readjustment steps without significant displacement*
		Linear	*Forward direction of travel at any velocity*
		Ball	*Dribbling with the ball*
		Lateral	*Shuffling of the feet to travel in a sideways direction*, *no crossover*
		Diagonal	*Crossover steps to travel in a diagonal direction*
		Rear	*Travelling directly backwards to the direction of facing*
		Rear plus	*Travelling backwards to the direction of facing with the addition of shoulder drops for readjustment*
		Deceleration	*Significant deceleration mechanics including landing*, *passing*, *tackling and receiving the ball*
Initiation	Starting position	Linear	*Typical initiation of an acceleration in a forward direction*
		Lateral	*The completion of a hip turn movement where the foot opposite to the intended sideways direction of travel steps over the other to set up for standard acceleration mechanics*
		Rear	*The completion of a drop step*, *followed by a hip turn to initiate standard acceleration mechanics in the direction opposite to the way the individual is facing*
	Change of direction	None	*No alteration to the current direction of travel*
		Lateral	*The completion of a side-step cut*, *where the foot opposite to the intended direction change is planted outside the centre of mass to initiate a sideways change of direction*. *Typically*, *short contact times*
		Front-Back	*The completion of a plant step where momentum in the forwards direction is stopped and a movement in the opposite direction is begun*. *Typically*, *longer contact times than cut stepping*
		Back-Front	*The completion of a plant step where momentum in the backwards direction is stopped and a movement in the opposite direction is begun*. *Typically*, *longer contact times than cut stepping*
Actualisation	Acceleration	Explosive	*A rapid acceleration including a sudden increase in leg turn over and overall velocity*
		Rolling	*A gradual acceleration where an increase in velocity is achieved over a more prolonged period*. *Less of a sudden increase in leg turnover*
	Maximum Velocity	Linear	*The upright running portion of the sprint is completed in a completely forward direction*
		Curved	*The upright running portion of the sprint is completed with the presence of any degree of curvature*. *Typically involves a lean of the torso towards the direction of the curve and the placement of the inside foot inside the centre of mass*
	Torso orientation	No rotation	*During upright running portion of the sprint the torso is kept facing directly forwards*
		Rotation	*During the upright running portion of the sprint is completed with the presence of any amount of rotation at the torso away from the direction of travel*
	Action during sprint	None	*No other action is performed during the sprint*
		Duel	*During the sprint there is presence of a dueling action with another body*. *An action that is not typically of standard sprinting mechanics*
		Ball	*During the sprint there is some ball involvement*, *including a pass*, *or dribbling with the ball where the individual kicks the ball and sprints after*
	Action at the End of Sprint	None	*The sprint does not end with any action*
		Duel	*The sprint ends with a dueling action*. *Including a tackle*, *or engagement of another body*
		Ball	*The sprint ends with an action including the ball such as a pass*, *shot*, *header or* *dribble*

It is argued that movement skill, pattern, and contextual information on sprinting to supplement the previously studied high-intensity running [16,17,27] would prove to be even more valuable for practitioners working in elite soccer. Due to the increased physical demands, potential performance benefits, and inherent injury risks associated with greater sprinting velocities [28], examination of specific movements associated with soccer sprinting would be beneficial [7,29,30]. A deeper insight into the movement skills and patterns associated with sprinting efforts could be used to assist in drill construction, testing battery selection, and inform physical preparation strategies for soccer players, enabling practitioners to achieve increased specificity and ecological validity [20,27,31]. Additionally, insight into the movement skills and patterns associated with sprinting could have important implications for preparing rehabilitating and previous injured athletes to the competitive sprinting demands of match-play [32]. Therefore, the aim of this exploratory analysis study was to quantify and contextualize sprinting actions of EPL soccer match-play with respect to the movement skills and patterns employed.

## 2. Materials and methods

### 2.1 Procedures and sample

Video match data of a single EPL soccer team utilised was secondary data taken from publicly available sources (3.2.6) (Premier League DVMS, ChyronHego). Data were treated confidentially, with ethical approval granted by the Manchester Metropolitan University ethics committee (ID: 45054), and written gatekeeper consent obtained from the club. Each of the analysed team’s EPL games from the 2017–18 season were assigned a reference number in ascending order correlating with the chronological order of the matches. 5 home and 5 away matches of the soccer team were then randomly chosen from these. These included matches against 9 separate opposition and involved 21 different players (average sprint evaluations per player 43 ± 33; average sprint evaluations per player per match 7 ± 3). Results of the matches included 3 wins, 4 draws, and 3 defeats. The team’s formation was classified as 4-5-1 on five occasions (456 sprint evaluations), 4-4-2 on three (257 sprint evaluations), and 5-3-2 on two (188 sprint evaluations). Whilst primary analysis was focused on all players, additional analysis was completed between playing positions. Positions were observed via two methods. Firstly, players were categorised as centre backs (CB), full backs (FB), central midfielders (CM), wide midfielders (WM), and central forwards (CF) [27]. Additionally, to support pitch location information, groupings were created based upon the playing positions’ location on the pitch: central (CB, CM, CF) and lateral (FB, WM).

From the 10 matches analysed, 901 total sprint efforts were recorded. A sprint was classified as the attainment of a velocity ≥ 7.0 m/s [3]. Whilst no official consensus exists, this is noted as the most widely employed velocity threshold in soccer analysis and would, therefore, be in line with previous research and practice [1]. Raw video-based locomotor coordinates were taken from official Premier League sources, Tracab (ChyronHego, USA). The raw data was then processed and filtered through a load management software to create velocity-time data (OpenField, Catapult Sports, Aus.). From this, time-stamps from the match clock were established for each effort classified as a sprint. These were then recorded for each player involved in the match. To classify these sprint efforts, official match video footage was obtained from the official Premier League DVMS online system (Premier League, UK) [33]; a portal database of all match footage powered by Hudl (Hudl, USA). Multiple camera angles were used for evaluation: tactical (high, wide-angle view from the centre of a lateral side of the pitch); 2) high behind (high angle, behind one of the goals); and broadcast (standard television broadcast view). Due to its ability to observe the most match-action, the tactical view was selected as the primary angle for analysis. If this view was obscured in any way, ‘high behind’ and ‘broadcast’ were respectively employed until the effort could be fully classified using the SMC. These matches averaged 12.8 ± 0.4 evaluated players per match. As no sprints were completed by goalkeepers over the match sample, each match involved 10 outfield players at one time. Sprint efforts of players who did not complete the full match were included and the substituted player into the match was treated as a like-for like replacement. Thus, 100% of the match time was analysed for 10 outfield positions.

### 2.2 Sprint movement classification

This exploratory analysis used a Football Sprint Movement Classification System (SMC) (Table 1) [34] adapted from the gamespeed model of Jeffreys et al. [26] to provide specific detail on how sprints are performed during soccer match-play. The system allows for match footage to be systematically and qualitatively analysed to comprehensively describe the movements completed whilst sprinting in soccer. Analysis of each sprint was completed across the three broad main categories of: 1) transition: the movements completed immediately prior to the sprint effort; 2) initiation: the movements associated with the beginning of the sprint effort; and 3) actualisation: the movements employed during the sprint effort (Tables 1 & 2). Match sprint time-stamps for each player during each match were ascertained and these were then systematically analysed according to previously established protocols with respect to transition, initiation, and actualisation (Tables 1 & 2) [34] by the lead researcher who is an experienced sports scientist and strength and conditioning coach. Excellent intra-rater reliability (*k =* 0.98, 97 sprint evaluations) was observed for a single match for the SMC, performed 7 days apart. Additionally, excellent inter-rater reliability (*k =* 0.95, 66 sprint evaluations) was demonstrated between the lead researcher and second rater who was an experienced sports scientist. An example sprint classification of a single effort is presented in S1 Table.

### 2.3 Statistical analysis

Data collected for each sprint was inputted into Microsoft Excel (Microsoft Corporation, Redmond, WA, USA). This data was then processed and formatted for analysis in a common statistical analysis software (SPSS, v26 Chicago, IL, USA). Processing included the establishing of means for each action category of each match, pooled across positions and by positional groups. Following the confirmation of normality utilising a Shapiro-Wilk’s test, a one-way analysis of variance (ANOVA) or independent t-test was completed to determine any statistical differences in the mean frequency of each within movement categories (transition, initiation, actualisation). Tukey HSD post hoc was utilised for multiple pairwise comparisons. Significance was set at *p* < 0.05. All data, unless otherwise stated, was presented as mean and standard deviation. Following this, Cohen’s *d* effect sizes (ES) were calculated to ascertain the magnitude of these differences. Magnitudes were classified as follows: trivial (<0.20), small (0.20–0.59), moderate (0.6–1.19), large (1.20–1.99) and very large (>2.0) [35]. Finally, the proportion of sprints within the SMC were calculated by positional groups and pooled across positions.

## 3. Results

Results for sprint data are pooled across positions presented by the system’s main categories of transition, initiation, and actualisation movements, and representative of average movements per match.

### 3.1 Transition movements

**Fig 1:**
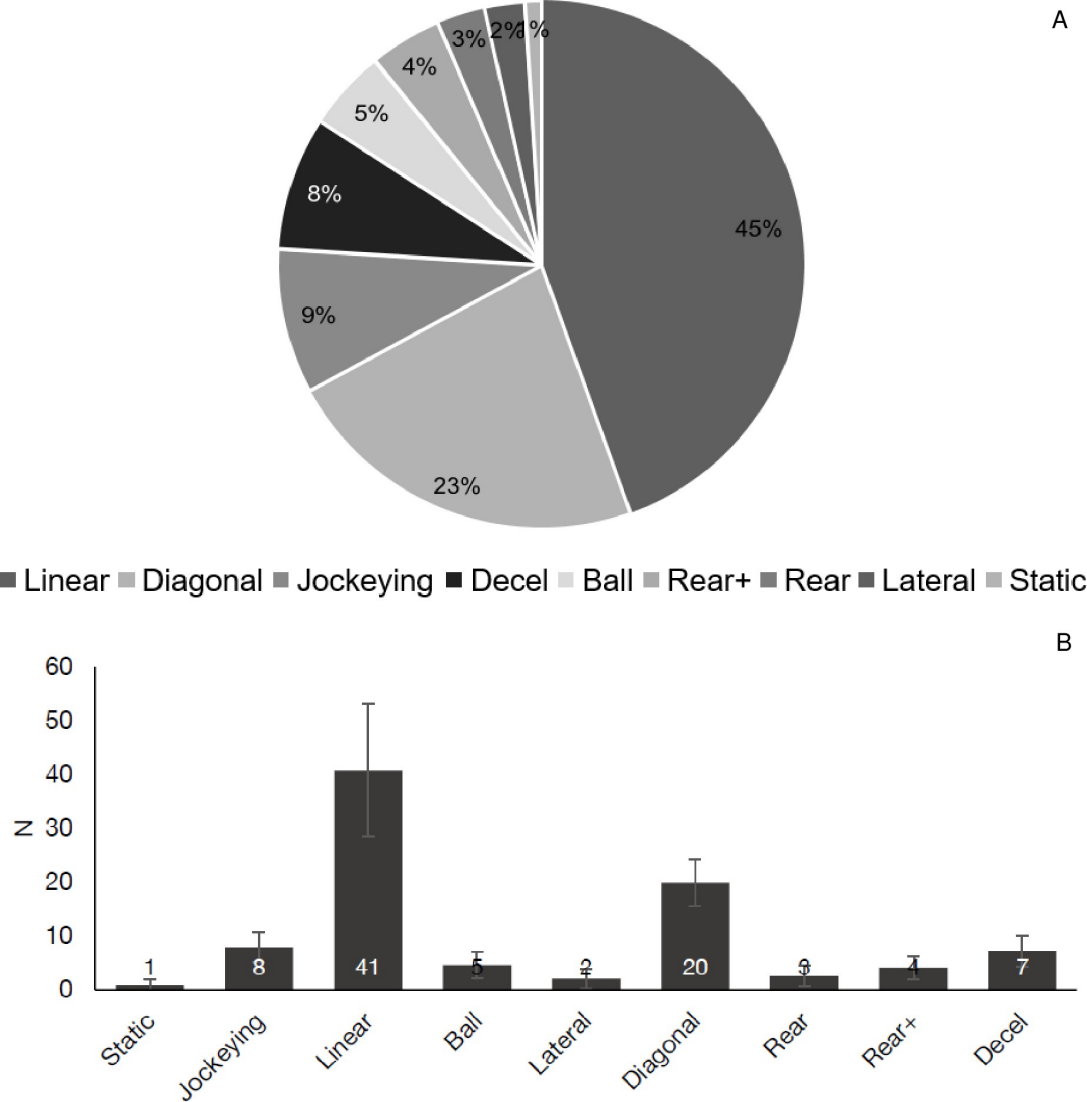
Transition movements observed during soccer match-play. (A) Average percentage of transition movements preceding sprints during match-play. B: Mean and Standard Deviation of the number of sprints completed during a match from different transition movements. Decel: Deceleration.

Significant differences were observed between the type of transition movements performed (*p* < 0.01) (Fig 1), with 68% of all efforts categorised as linear (45%) or diagonal (23%) (Fig 1). Linear transition movements preceding sprints (41 ± 12) occurred significantly, very largely more frequently than all other movements (*d* = 2.1–4.3, *p* < 0.01), followed by diagonal (20 ± 4) which occurred significantly, very largely more frequently than all other movements excluding linear (*d* = 3.1–5.7, *p* < 0.01). No other significant differences were observed across transition movements (*p* > 0.05); however, the least frequent movement preceding a sprint during match-play was static (1 ± 1), accounting for only 1% of efforts. All remaining categories (excluding linear and diagonal) accounted for less than 10% of sprint efforts each, with on average 8 ± 3 (9%) jockeying and 7 ± 3 (8%) deceleration transition movements observed (Fig 1).

### 3.2 Initiation movements

**Fig 2:**
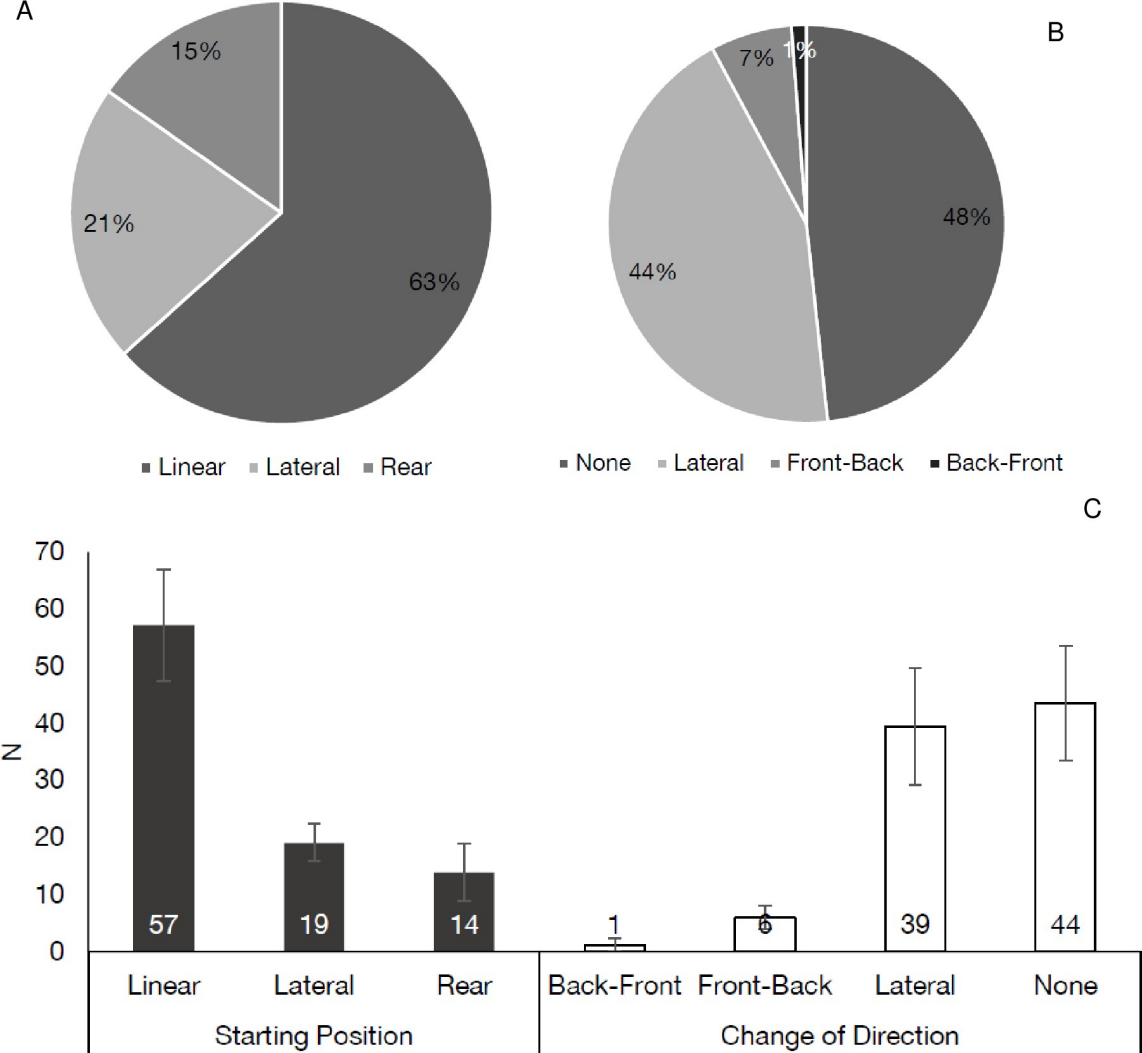
Initiation movements observed during soccer match-play. (A) Average percentage of different starting positions utilised during match-play. (B) Average percentage of different change of direction initiations utilised during match-play. (C) Mean and Standard Deviation of the number of sprints completed during a match from different initiation movements.

Significant differences were observed between the type of sprint initiation movements performed (*p* < 0.01) (Fig 2), with 63% of all efforts initiated from a linear starting position, followed by lateral (21%) and rear (15%). When observing sprints with COD initiations, no COD was the most frequent (48%), followed by lateral (44%), front-back (7%), and back-front (1%) (Fig 2). Linear sprint starting positions (57 ± 10), occurred significantly, very largely and approximately three times more often than other initiation movements (*d =* 5.0–5.3, *p* < 0.01). Conversely, no significant differences (*d =* 1.2, *p* > 0.05) were observed between lateral (19 ± 3) and rear (14 ± 5) starting positions (Fig 2) but a large effect size was observed.

Most sprint initiations occurred with no COD prior to the effort (Fig 2), with no COD (44 ± 11, *d* = 4.9–5.6, *p* < 0.01) and lateral COD (39 ± 11, *d =* 4.3–4.9, *p* < 0.01) occurring significantly, very largely more frequently than back-front (1 ± 1) and front-back (6 ± 2) which were rarely performed (Fig 2). However, when all COD efforts were pooled, most sprints were initiated with a type of COD compared to none (52% vs 48%) (Fig 2).

### 3.3 Actualisation movements

**Fig 3:**
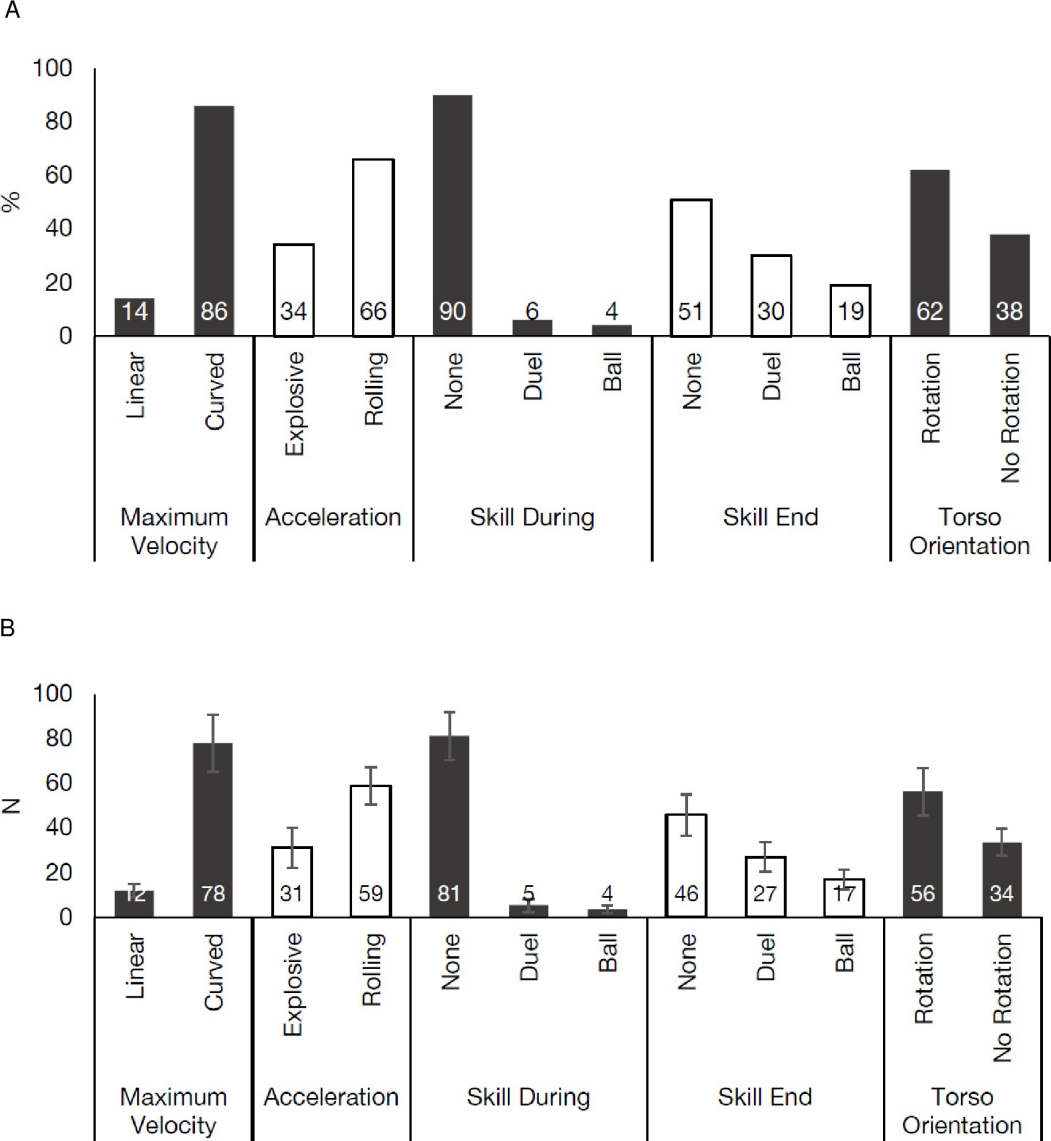
Actualisation movements observed during soccer match-play. (A) Average percentage of different actualisation movements during match-play. (B) Mean and Standard Deviation of the number of sprints completed during a match from different actualisation movements.

Significant differences were observed between the sprint actualisation movements performed during match-play (*p* < 0.01) (Fig 3), with curvilinear sprint efforts (78 ± 13; 86%) during the maximum velocity phase significantly, very largely occurring (*d* = 7.2, *p* < 0.01) more frequently than linear efforts (12 ± 3; 14%). Rolling accelerations (59 ± 8; 66%) were significantly, very largely (*d* = 3.2, *p* < 0.01) more frequent than explosive acceleration actions (31 ± 9; 34%). During the sprint effort, no action (81 ± 11; 90%) was the most frequently observed, which occurred significantly, very largely more frequently than duel (5 ± 3; 6%) and ball (4 ± 2; 4%) (*d* = 9.1–9.5, *p* < 0.01). No significant differences were observed between duel and ball (*d* = 0.7, *p* > 0.05) with a moderate effect size. At the end of the sprint efforts, no action was the most frequent (46 ± 10; 51%), occurring significantly, very largely more frequently than both duel (27 ± 7; 30%) and ball (17 ± 4; 19%) (*d* = 2.2–3.8, *p* < 0.01). Additionally, duel was significantly more frequent than ball at the end of the efforts (*d* = 1.7, *p* < 0.05) with a large effect size. However, when combined, 49% of sprints in soccer ended with a type of action (i.e., duel or ball) (Fig 3). Finally, the majority of sprint efforts were completed with torso rotation (56 ± 11; 62%) away from the direction of travel, significantly, very largely greater than no rotation (34 ± 6; 38%) (*d* = 2.7, *p* < 0.01).

### 3.4 Summary of pooled sprint data

The most common average actions within each category are displayed in Table 3. The majority of sprint efforts involve linear starts from linear transition movements and with no COD. These efforts are most commonly performed with a rolling acceleration. Whilst sprinting, efforts typically possess some amount of curvature and torso rotation. In addition, the majority of efforts are completed without any skill during or after.

**Table 3:** Summary of the of the most common sprints action within each sub-category of the football sprint movement classification system.

Sub-category	Most frequent action	Average percentage
**Transition Movement**	Linear	45%
**Starting Position**	Linear	63%
**Change of Direction**	None	48%
**Maximum Velocity**	Curved	86%
**Acceleration**	Rolling	66%
**Skill During**	None	90%
**Skill End**	None	51%
**Torso Orientation**	Rotation	62%

### 3.5 Sprint positional comparisons

**Fig 4:**
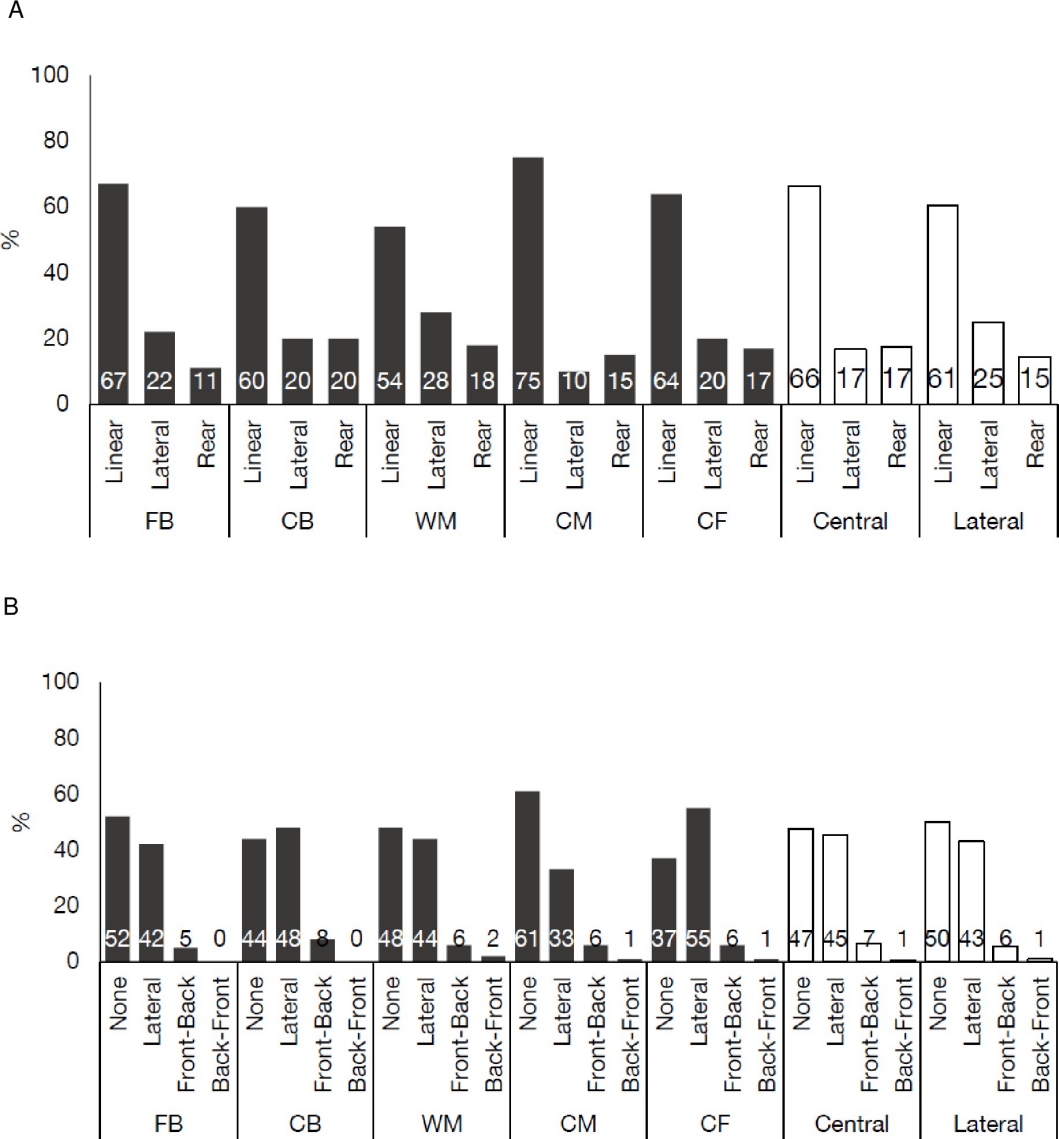
Sprint Initiation movements observed during soccer match-play by playing position and location. (A) Average percentage of different initiation movements during match-play by playing position. (B) Average percentage of different initiation movements with or without a COD during match-play by playing position. CB: Centre backs; FB: Full backs; CM: Central midfielders; WM: Wide midfielder; CF: Central forwards.

Transition movement average percentages by positional group are presented in S1 Data. When observing initiation movements by playing position, CM were found to compete the greatest proportion of their sprints from a linear starting position (75%), whilst WM the least (54%) (Fig 4). Conversely, WM completed the greatest proportion of sprints from a lateral starting position (28%), with CM the lowest (10%). CB completed the greatest proportion of sprints from a rear starting position (20%), with FB the least (11%). Of the five playing positions, three of these completed the greatest proportion of their sprints from no COD (FB = 52%; WM = 48%; CM = 61%) (Fig 4), whereas lateral COD for CB (48%) and CF (55%) was the most common. Across positions, CF completed the smallest proportion of sprints with no COD compared to all others (37%). Although Back-Front and Front-Back COD initiations were generally rare, 8% of CB sprints began with a front-back direction change; the most of all positions (Fig 4).

**Fig 5:**
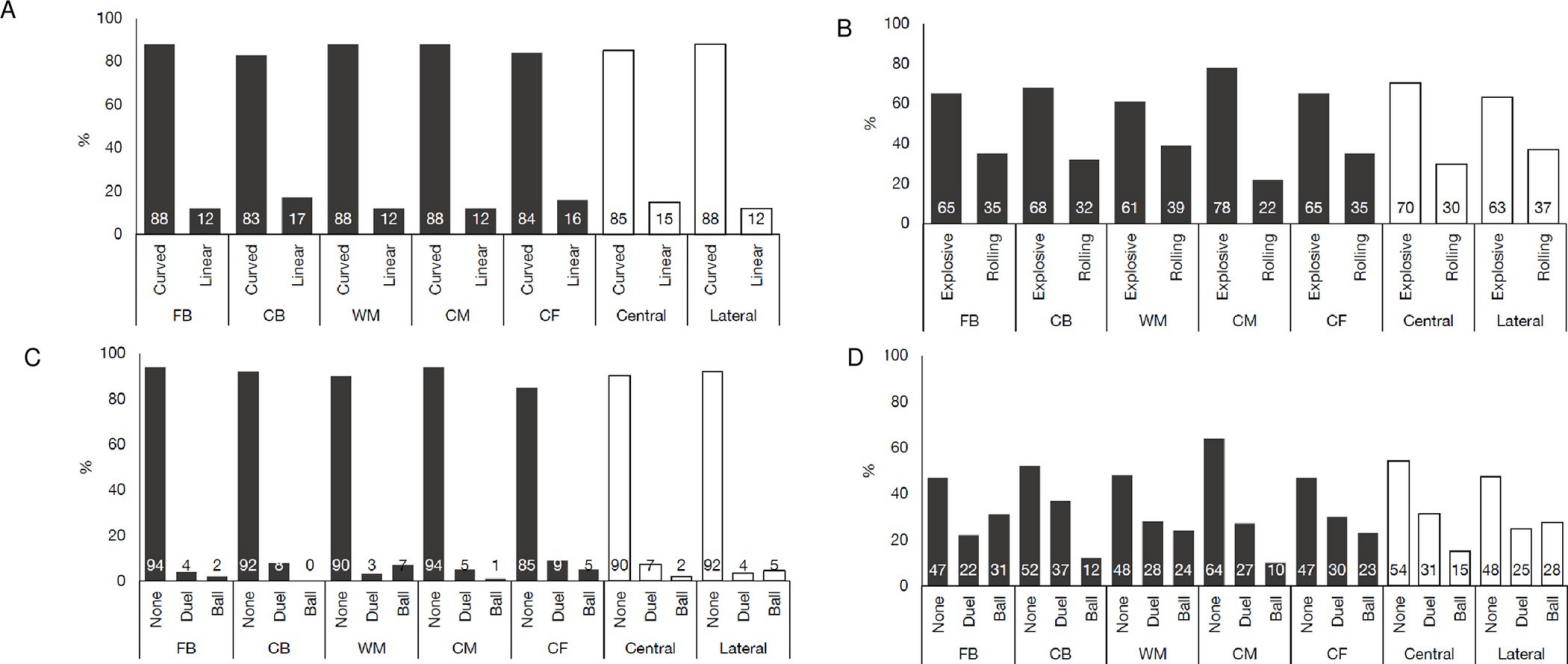
Sprint actualisation movements observed during soccer match-play by playing position and location. (A) Average percentage of curved or linear sprints during match-play by playing position. (B) Average percentage of explosive or rolling accelerations during match-play by playing position. (C) Average percentage of sprint actions during the effort during match-play by playing position. (D) Average percentage of sprint actions at the end of the effort during match-play by playing position. CB: Centre backs; FB: Full backs; CM: Central midfielders; WM: Wide midfielder; CF: Central forwards.

When observing actualisation movements by playing position, all position groups completed most of their sprints with curvature (84–88%), rather than strictly linear (12–16%). Additionally, most sprints were performed with a rolling acceleration (61–78%) compared to explosive across positional groups (Fig 5), with CM completing the greatest proportion (78%) whereas WM performing the lowest (61%). During the sprint effort, across all playing positions the most common action was no action (85–94%) (Fig 5). Similarly, for all playing positions, the greatest proportion of sprints ended with no action (47–64%), though 10–36% sprint actions ended with duels or ball. Finally, all positions completed most sprints with torso orientation (Fig 5), with WM performing the lowest (57%) and FB the greatest (72%).

## 4. Discussion

The aim of this study was to quantify and contextualize sprinting actions of EPL soccer match-play with respect to movement skills and patterns employed. The primary findings were that sprints during EPL match-play were initiated from, and performed with, a variety of different movement skills and patterns in relation to different sport-specific outcomes (Table 3, Figs 1–5). Whilst variation in sprinting movement patterns were evident (Figs 1–5), specific sprinting actions with certain movement characteristics occur more frequently than others (Table 3, Figs 1–5). Generally, our results show that sprints during EPL soccer regularly begin from a linear initiation position (63%), without a COD (48%) and from forward travelling (linear and diagonal) transition movements (68%) (Table 3). These sprints are often attained from a rolling acceleration (66%), performed with a degree of curvature (86%) and torso rotation (62%), and typically ending with an action such as dueling with an opponent or involvement with the ball (49%) (Table 3). Additionally, the sprint movements characteristics slightly differed across playing position (Figs 4 and 5). Consequently, this study confirms that sprints during EPL soccer matches are initiated from and performed with a variety of different movement skills and patterns in relation to different sport-specific outcomes. The observations and characterization of the specific movement skills and patterns during sprinting can therefore serve as a reference point to assist in the development of more effective physical preparation programmes, inform position-specific contextualized sprinting drills to achieve better specificity and potential transfer of training, while also informing speed testing protocols.

The current study shows that whilst sprinting in soccer consists of a variety of different movements (Figs 1–5), there is a clear tendency for sprints to occur from what could be classed as ‘typical’ or ‘traditional’ positions (i.e., linear and sagittal plane dominant). These are positions that would resemble more classically defined sprints, such as those observed in Track and Field. Within the categories of transition, COD, and initiation, those classed as linear or forwards tend to be the most commonly observed (Fig 1, Table 3). This finding is in line with a previous study on high-intensity efforts, where the majority of actions were preceded by turns of less than 90° [27]. It would be reasonable to assume, therefore, based on the angle-velocity trade-off, that these types of efforts are faster, induce lower physiological and biomechanical loading than efforts requiring greater deflections of the centre of mass and varying initiation positions [20,36]. The current study is unable to wholly distinguish the cause of this prominence of ‘typical’ sprinting movements in soccer. The constraints, sport-specific stimuli, tactical scenarios, and overall visual scanning requirements (i.e., game patterns, tactics, location of attackers, defenders, ball, and environment) imposed during the match may influence this outcome [20,25], or the individual’s skill level may mean they perceive affordances (i.e., opportunities for action) differently which leads to different action outcomes that may have been seen elsewhere [15,26]. As such, future research is needed to better understand how physical, tactical, and technical performance interrelate (i.e., full integration) with regards to contextual sprinting (i.e., how and why) in soccer [16,18].

In the present study, not all sprints were initiated from linear or ‘typical’ positions, with ~30–50% of efforts lateral, rear, or involving a COD (Fig 2), whereby these differences become more evident when broken down by playing position (Fig 4). For example, WMs initiated 46% of their sprint efforts from non-linear positions, compared to 25% for CMs (Fig 4). It is therefore reasonable to assume that the match, a player’s position within the match, a team’s strategy, and the subsequent tactical demands required of a player dictate the types of movements that present themselves during sprinting [37], as recently observed for high-intensity running during soccer which provided insight into the physical-tactical requirements [16,17,27]. Previously researchers have shown that higher level soccer players sprint less during a match than their lower-level counterparts [38]. These higher-level players appear to be more selective with their high-intensity efforts, potentially conserving their energy for the most crucial moments of match-play. It could therefore be potentially the case that the highly-skilled players in the current study attempt to ‘default’ to these linear-type efforts when possible as a means of efficient movement to accelerate and attain higher velocities [20,36]. Yet, as noted, it is currently unclear what is the true cause of this variation (i.e., why these sprint actions occur), and it is likely that specific match related tasks are the cause of this variation [15]; thus, it is a logical progression of the research to next begin to quantify these tasks.

Whilst the transition into and initiation of sprint efforts are broadly split between what are described as typical linear efforts and non-linear, the actualisation of the sprint effort itself appears to be rarely typical in the present study (Fig 3). During the sprint effort, non-linear (i.e.., curved) sprints were predominantly exhibited (87%), with torso rotation commonly observed (64%) (Figs 3 & 5). This is similar to the observations of previous research in high-intensity effort classifications which observed swerves and arc runs to be the most commonly occurring action [27], while curved sprints ≤ 30° have also been observed during youth-team soccer match-play [19]. Additionally, Brice et al. [39] reported curved motions of travel in EPL soccer ranging from 3.5–11 m radii, while seminal time-motion analysis from Bloomfield et al. [21] found soccer players perform ~10–20 swerves during match play, but provided limited information as to how these actions were classified. Curved sprints, in contrast to CODs, offer the advantage of deviating the path of travel while attaining or maintaining high sprinting velocities [19,20]. It is worth noting that curved sprinting is biomechanically different to linear sprinting, with clear differences in kinetics, kinematics, spatio-temporal characteristics, and muscle activity which are further influenced by the curvature (radius) [20,23,40]. Moreover, the inside and outside leg also display neuromuscular and kinetic and kinematic differences, with the inside leg serving a frontal plane stabilising function, whereas the outside leg primarily has a propulsion and rotational stabiliser function [20,24,41].

Although it was beyond the scope of the current study to explore the tactical and technical context why curved sprints occurred, soccer players may perform these in- and out-of possession, such as in ‘overlapping’ situations, maintaining an onside position, and performing ‘recovery’ and ‘covering’ runs [21,27,42,43]. Additionally, the finding that sprints were generally performed with trunk rotation is noteworthy, and could have important injury implications, due to the propensity to generate greater mechanical loads at the knee with trunk rotation compared to linear running [44], while trunk rotation has been recently identified as a visual characteristic observed during non-contact running hamstring strain injury inciting events in rugby union [29]. With this in mind, isolated, traditional track and field based sprinting programmes may not be the most effective training modality in this context, and thus practitioners should ensure their athletes have the physical capacity to tolerate the unique imposed loading associated with curved sprinting [45]. Nevertheless, the ability to maintain velocity whilst travelling along a curvilinear path (and/or dissociating the torso) appears to be a key skill for soccer players [8], but further detail is required to elucidate the exact nature of these efforts and how to best train these qualities [43].

Observed movement is constrained by the interplay between the task, environment, and the organism [37,46]. Thus, with the present study providing a greater understanding of the sprint movements that occur in soccer, training drills that seek to mimic these can be designed more effectively for primary transfer of training [47]. Through a constraints-led approach, drill design can be focused on creating ‘repetition without repetition’, where the athlete is exposed to tasks that allow for exploration and promote movement degeneracy and diversification through the seeking of movement solutions [25,37,46,48]. The constraints to these representative drills can be manipulated; for example, by introducing contextual interference, altering starting positions, manipulating stimuli (i.e., location of attackers, defenders, and ball), and reducing or increasing the pitch size to elicit differing responses and learning experiences [45,49,50]. Similarly, practitioners possessing enhanced knowledge of the specific movements associated with sprinting during soccer are better placed to successfully rehabilitate players to cope with the physical demands when returning to match-play [15,51]. While the present study has highlighted the most frequently occurring sprint movement skills and patterns displayed during soccer, practitioners should not mistake frequency for importance, and thus further research regarding the tactical and performance implications of sprints during soccer match-play requires further investigation. Nevertheless, this study has identified that soccer players perform a plethora of transition, initiation, and actualisation movements when sprinting (Figs 1–5). Therefore, it is likely beneficial that soccer players are proficient and possess the ability to perform a range of different sprint skills and movement patterns, and possible movement solutions, so they have the adaptability to meet the chaotic and unpredictable tactical and technical demands of soccer match-play [20,25,45].

It should be noted that the sprint data of the present study is representative of only one team from one EPL season. Due to this, the ultimate, direct applicability of the results are limited to the team used as key factors such as formation, team strategy, match location, quality of opponent, and athlete physical capacity are likely to affect the eventual movement outcomes presented [52–54], and should be considered in future research. Thus, caution must be taken when extrapolating these to other teams, playing standards, ages, and sexes. However, within context of these limitations, the results presented will be pertinent to soccer within the utilised population of EPL soccer. To resolve this potential limitation, future research should seek to replicate the current study using a controlled variety of teams, and potentially seek to draw comparisons across different formations, playing strategies, match location, and playing level. For example, a team instructed to ‘press’ often will likely present a different sprint movement profile to a team which remains deeper in their formation. Moreover, it should be noted that sprint movement profiling was qualitative, and the exact nature of curvilinear motion (i.e., specific angles, distances and the bioenergetic cost of curved sprint efforts) or torso dissociation was not quantified, nor were body impacts quantified which have important implications for exercise volume, tissue damage, and neuromuscular fatigue [55–57], and is thus a future avenue for further research. Finally, as previously stated, this study did not quantify the pitch location of tactical scenarios by which sprinting occurred which requires further exploration. Recently researchers have provided insights into high-intensity (≥ 5.5 m/s) running contextualised periods of play [16], whilst examining generalised and specialised tactical roles [17]. Thus, similar research is required to further contextualise higher velocity sprinting actions.

## 5. Conclusion

The primary findings were that sprints during EPL soccer matches are initiated from and performed with a variety of different movement skills and patterns in relation to different sport-specific outcomes (Table 3, Figs 1–5). For the first time, the current study presents practitioners with an intricate understanding of how sprinting (i.e., skills and movement patterns) in EPL soccer are completed. Specifically, curvilinear sprints appear to be a frequently occurring action during soccer match-play. As such, isolated, traditional track and field based sprinting programmes may be sub-optimal in the physical preparation of soccer players, and that practitioners could now use the current findings to refine their practice to better reflect the physical demands of match-play and potentially achieve better training specificity and transfer. For example, curvilinear sprint training and the assessment of this quality (e.g., use of the penalty arc and electronic timing gates) are advocated for practitioners working in soccer. Specifically, practitioners can consider incorporating sprint drills which are initiated from a rolling start, with a moderate volume of sprints which include some form of torso dissociation or rotation to better reflect the unique sprinting demands of soccer. Finally, practitioners should also develop their soccer players’ ability to perform sprints from various transition (e.g., linear, lateral, jockeying; Table 2) and initiation movements (e.g., linear, lateral, rear; Table 2) to develop their player’s movement solutions and physical literacy.

## Supporting information

S1 Table An example sprint classification of a single effort. (DOCX)

S1 Data Sprint transition results with positional comparisons. (DOCX)

S2 Data Raw data containing contextual sprint classification data. (XLSX)
