# Supplementary material for: A classification of specific movement skills and patterns during sprinting in English Premier League soccer
Source: PLoS One. 2022 Nov 11;17(11):e0277326. doi: 10.1371/journal.pone.0277326 (PMC9651586; doi:10.1371/journal.pone.0277326)
Supplement: S1 Table — (DOCX) [file pone.0277326.s001.docx]

| **Supplementary material 1**. An example sprint classification of a single effort is presented in supplementary material 1 | | |
| --- | --- | --- |
| **Main category** | **Sub-category** | **Action** |
| **Transition** | Transition Movement | Deceleration. |
| **Initiation** | Starting Position | Lateral |
|  | Change Of Direction | Lateral |
| **Actualisation** | Acceleration | Explosive |
|  | Maximum Velocity | Linear |
|  | Torso Orientation | Torso rotation |
|  | Action During | None |
|  | Action End | Ball |
