# Supplementary material for: A classification of specific movement skills and patterns during sprinting in English Premier League soccer
Source: PLoS One. 2022 Nov 11;17(11):e0277326. doi: 10.1371/journal.pone.0277326 (PMC9651586; doi:10.1371/journal.pone.0277326)
Supplement: S1 Data — (DOCX) [file pone.0277326.s002.docx]

**Supplementary material 2**

When observing Transition Movements completed by Playing Position, large differences are seen (Table 1). All positions completed the majority of their sprints from Linear movements, followed by Diagonal. As discussed, these are the most ‘typical’ forward-moving transition movements. When grouped together, CM were seen to complete the greatest proportion of sprints from Linear and Diagonal movements (76%), whereas WM completed the least (55%).

**Table 1.** Average percentage of sprints completed during a match from different Transition Movements, categorised by playing position.

Beyond Linear and Diagonal (the most frequent for all positions), the next most common movements utilised were: FB - Jockeying and Rear (7%), CB - Rear+ (9%), WM - Jockeying (14%), CM - Deceleration (8%), and CF - Jockeying (11%). Thus, whilst the most common movements are similar across positions, differences exist beyond this. This could have potential implications for how specific positions are prepared for match play.

Certain positions appear to use more varied Transition Movements than others. As noted, 45% of WM sprints are completed from positions that are not typical forward direction movements (Linear and Diagonal). This is particularly different from CM where only 14% are from non-forward direction movements (Table 4.5). This is further evident by CB recording 0 sprints in two of the categories (Ball and Rear). FB and WM were the only two positions to record sprints within all movement categories. These positions are both categorised as Lateral playing locations.

Lateral categorised positions complete 38% of sprints from non-forward direction Transition Movements, as opposed to only 28% for Centrally located positions. This is predominantly due to 12% less Linear movements for the Lateral positions. Lateral positions notably complete more sprints from Jockeying and Ball Transition Movements (+4% and +4%). These differences are likely due to constraints such as the positions location on the field and the unique tactical demands.
